# Survival of Civilian and Prisoner Drug-Sensitive, Multi- and Extensive Drug- Resistant Tuberculosis Cohorts Prospectively Followed in Russia

**DOI:** 10.1371/journal.pone.0020531

**Published:** 2011-06-10

**Authors:** Yanina Balabanova, Vladyslav Nikolayevskyy, Olga Ignatyeva, Irina Kontsevaya, Clare M. Rutterford, Anastasiya Shakhmistova, Nadezhda Malomanova, Yulia Chinkova, Svetlana Mironova, Ivan Fedorin, Francis A. Drobniewski

**Affiliations:** 1 Queen Mary College, Barts and the London School of Medicine, University of London, London, United Kingdom; 2 Samara Oblast Tuberculosis Dispensary, Samara, Russia; St. Petersburg Pasteur Institute, Russian Federation

## Abstract

**Objective and Methods:**

A long-term observational study was conducted in Samara, Russia to assess the survival and risk factors for death of a cohort of non-multidrug resistant tuberculosis (non-MDRTB) and multidrug resistant tuberculosis (MDRTB) civilian and prison patients and a civilian extensive drug-resistant tuberculosis (XDRTB) cohort.

**Results:**

MDRTB and XDRTB rates of 54.8% and 11.1% were identified in the region. Half (50%) of MDRTB patients and the majority of non-MDRTB patients (71%) were still alive at 5 years. Over half (58%) of the patients died within two years of establishing a diagnosis of XDRTB. In the multivariate analysis, retreatment (HR = 1.61, 95%CI 1.04, 2.49) and MDRTB (HR = 1.67, 95%CI 1.17, 2.39) were significantly associated with death within the non-MDR/MDRTB cohort. The effect of age on survival was relatively small (HR = 1.01, 95%CI 1.00, 1.02). No specific factor affected survival of XDRTB patients although median survival time for HIV-infected versus HIV-negative patients from this group was shorter (185 versus 496 days). The majority of MDRTB and XDRTB strains (84% and 92% respectively) strains belonged to the Beijing family. Mutations in the *rpoB* (codon 531 in 81/92; 88.8%), katG (mutation S315T in 91/92, 98.9%) and *inhA* genes accounted for most rifampin and isoniazid resistance respectively, mutations in the QRDR region of *gyrA* for most fluroquinolone resistance (68/92; 73.5%).

**Conclusions:**

Alarmingly high rates of XDRTB exist. Previous TB treatment cycles and MDR were significant risk factors for mortality. XDRTB patients' survival is short especially for HIV-infected patients. Beijing family strains comprise the majority of drug-resistant strains.

## Introduction

Multidrug-resistant tuberculosis (MDRTB) is a growing threat to national tuberculosis (TB) programmes world-wide. Extensive drug-resistance (XDR), i.e. additional resistance to fluoroquinolones (FQ) and injectable agents decreases chances of survival and success further [1], [2], [3], [4], [5], [6], [7]. So far co-infection of HIV and XDRTB has been largely fatal [2], [8], [9], [10], [11].

The problem of drug–resistant tuberculosis is particularly acute in the countries of the Former Soviet Union (FSU) where almost half of all TB cases are resistant to at least one drug and the MDRTB rate among new cases exceed 20% in some countries [12]. The extent and pattern of resistance to second-line drugs (SLD) among MDRTB isolates remain unknown in most FSU countries due to insufficient laboratory infrastructure and testing. Only limited data is available on patient survival in the long term and where reported in Russia, cure rates and short-term survival have been poor regardless of HIV status [13], [14].

In Samara Oblast (a region of the Russian Federation with a population of 3.3 million) high rates of MDRTB (20% among new and 43% among previously treated cases) are coupled with a fast growing HIV epidemic (HIV-positive cases reaching 43 000 in December 2010) [15], [16]. A drug-resistance survey conducted in 2001–2002 in this region demonstrated relatively low rates of resistance to second-line drugs with no XDRTB cases [17]. Prolonged hospitalization (minimum 3 months) is compulsory for culture-confirmed patients; HIV-infected patients stay at the same hospitals with other patients including drug-resistant cases. Institutional infection control measures are not fully implemented, i.e. there are no negative-pressure rooms, appropriate protective masks are not worn regularly by staff, there is no strict separation of in-patients with sensitive and resistant tuberculosis and there are opportunities for patients to meet socially. The potential for HIV infected patients to become infected with drug resistant TB is high.

The aim of this study was to determine the long-term survival of drug-sensitive (non-MDR), MDRTB and XDRTB patients in the civilian and prison sectors, and to identify risk factors including TB strain type, associated with survival.

## Materials and Methods

### Ethics statement

The study was approved by Samara Medical University Ethics Committee and received a waiver of informed consent.

### Study settings and population

Results of two independent patient cohorts from the same region of Russia are presented. Patients from a non-MDR/MDRTB Cohort (N = 880) were prospectively consecutively recruited from the civilian and prison sectors into the study in 2002–2003 within a pilot DOTS-programme; this “2002-3 TB Cohort” was comprised of new (88%) and relapse cases infected with MDRTB and non-MDR strains (including fully-sensitive and mono-/polyresistant) and was followed prospectively until December, 31^st^ 2008 to give long-term survival data.

XDRTB Cohort consisted of all civilian patients diagnosed with XDRTB in the same region in 2008 (“2008 XDRTB Cohort”). All identified XDRTB patients were enrolled into the analysis (n = 92). Basic data on history of TB and survival were collected using routine TB patients' register supplemented with detailed epidemiological and clinical data from chart review.

### Bacteriological methods

For all patients, smear microscopy was performed using the Ziehl-Neilsen method with culture on Lowenstein-Jensen media according to standard procedures [18]. Overall, mycobacterial isolates were obtained from 783 patients in the 2002-3 Cohort.

First-line drug susceptibility testing (FLD DST) was performed using either conventional solid media (by the absolute concentration method), or the MGIT 960 system using standard techniques [18], [19]; second line DST was performed using the MGIT system as described [20], [21]. Drug concentrations used for second-line drugs were (µg/ml): Ofloxacin (Ofl): 2.0; Moxifloxacin (Mox): 0.25; Amikacin (Amk): 1.0; Capreomycin (Cap): 2.5; Prothionamide (Pt): 2.5 [20].

Prior to routine implementation of SLD MGIT 960 testing, laboratory staff were trained, and the methodology quality controlled and validated by the UK Health Protection Agency National Mycobacterium Reference Laboratory (HPA NMRL); for quality control purposes 31 isolates were randomly selected and re-tested additionally for FLD and SLD drugs with complete concordance at the NMRL.

### Molecular genotyping methods

Detection of mutations in genes associated with resistance to rifampin and isoniazid was performed on all isolates from both Cohorts using an in-house reverse hybridization assay [22], [23], [24] capable of detecting the most common mutations in genes *katG, inhA,* and *rpoB*.

Identification of mutations associated with the FQ resistance was performed for all XDRTB isolates at the NMRL by sequencing the quinolone-resistance-determining region (QRDR) of the *gyrA* gene (codons 88–100) using a pyrosequencing assay (Qiagen, Hilden, Germany). Genetic families were identified by spoligotyping for both Cohorts [25]. XDRTB isolates were also genotyped at the Samara Oblast Dispensary Laboratory and at the NMRL, London using Variable Number Tandem Repeat (VNTR) typing with 15 MIRU loci [26] and an additional panel of 13 highly discriminatory loci [27], [28]. PCR fragments were separated and sized manually (in Samara) and by using automated capillary electrophoresis in London (CEQ8000 Genetic Analysis System, Beckman Coulter) as described previously [25].

### Main outcome measures

All cases within both Cohorts were included in the descriptive analysis.

The study endpoint was time to death, calculated since TB diagnosis in the 2002-3 TB Cohort patients and from XDRTB identification in the 2008 XDRTB Cohort patients. Survival time was the difference between the diagnosis and death dates. Cases who died before therapy started from the 2002-3 TB Cohort (n = 3) were not included. Cases who were alive were censored: for the 2002-3 TB Cohort-at 31^st^ of December 2008, for the 2008 XDRTB Cohort- at 31^st^ of December 2009.

In the 2002-3 TB Cohort potential predictors of survival collected were: gender, age, residence (civilian, prisoner), infection type (new, relapse), HIV status (negative, positive), strain family (Beijing, non-Beijing) and resistance (MDRTB, non-MDRTB). A proportion of patients (n = 97) which were not culture-confirmed (diagnosis was based on clinical/radiological presentation) were excluded from the analysis of the effect of drug-resistance and strain type on survival.

In the 2008 XDRTB Cohort potential predictors of survival collected were: gender, age, residence (civilian, prisoner), infection type (new, re-treatment), HIV status (negative, positive), extent of lung damage (number of lung zones affected and presence of cavities), clinical symptoms (cough, shortness of breath (SOB), fever, weight loss, chest pain, haemoptysis), strain family (Beijing, non-Beijing), adherence to treatment and alcohol/drug abuse.

Although recruitment occurred in the same settings, the two groups were analysed independently to avoid bias that may result from combining them.

### Statistical analysis

All data was entered on a password protected stand-alone database to maintain confidentiality.

Statistical analysis was carried out separately for both Cohorts using STATA 10.0 (Stata-Corp, TX, USA).

The χ2-test was used to compare proportions. Tests of significance were 2-sided, and P = 0.05 was considered statistically significant. Survival data was summarised using Kaplan-Meier graphs. Univariate analysis using Cox's proportional hazards model was performed for each potential predictor. Those which were significantly associated with increased risk of death at the 10% significance level were fitted together in a multivariate model. Analysis was performed on the available case sample (i.e. that each analysis only included participants where all their information required for that analysis was present). If a participant was lost to follow up they were censored at the last time they were known to have been alive.

The variables included in the final model were checked for the assumption of proportional hazards and the proportional hazards assumption was not violated (data not shown).

Statistical analysis was conducted by an author independent of the recruitment or laboratory sites.

## Results

### Rate of MDRTB and XDRTB in population

From a comprehensive analysis, in 2008 of 2048 individual patients (from consecutive patients at all 36 clinics) an overall rate of MDRTB of 54.8% (1123/2048) was obtained; SLD was performed on, and readable results obtained from 1025/1123 (91.3%) MDRTB isolates, and 114 XDRTB (11.1%) isolates were identified. In 2002-3 TB Cohort the rate of MDRTB was 17.5% among new patients and 34.0% among relapse cases [29].

### Clinical and demographic characteristics

The main clinical and demographic characteristics of both Cohorts are given in Table 1.

**Table 1:** Core common clinical and demographic characteristics of patients from the 2002-3 TB Cohort and 2008 XDRTB Cohort*.

Characteristic, no.(%)	Categories	2002-3 TB Cohort				2008 XDRTB Cohort
		DST not performed N = 97	MDR N = 139	Non-MDR N = 644	Total N = 880	XDR N = 92
**Gender**	Male	97 (100%)	119 (86%)	493 (77%)	709 (81%)	68 (74 %)
	Female	0 (0%)	20 (14%)	151 (23%)	171 (19%)	24 (26%)
**Age**	≤40 y.o.	85 (88%)	80 (58%)	321 (50%)	486 (55%)	45 (49%)
	>40 y.o.	12 (12%)	59 (42%)	323 (50%)	394 (45%)	47 (51%)
**Imprisonment**	Civilian	0 (0%)	106 (76%)	610 (95%)	716 (81%)	92 100%)
	Prisoner	97 (100%)	33 (24%)	34 (5%)	164 (19%)	0
**Treatment history**	New	91 (94%)	122 (88%)	590 (92%)	803 (92%)	5 (6%)
	Re-treatment	6 (6%)	17 (12%)	52 (8%)	75 (9%)	85 (94%)
	Total	97	139	642	878	90
**HIV Status**	Negative	84 (87%)	133 (96%)	625 (97%)	842 (96%)	80 (91%)
	Positive	13 (13%)	6 (4%)	19 (3%)	38 (4%)	8 (9%)
	Total	97	139	644	880	88
**Strain family**	Beijing	-	77 (84%)	193 (43%)	270 (50%)	85 (92%)
	Other	-	15 (16%)	251 (57%)	266 (50%)	7 (8%)
	Total	-	92	444	536	92
**Survival status**						
	Alive	80 (82%)	98 (71%)	490 (76%)	668 (76%)	39 (42%)
	Dead	17 (18%)	41 (30%)	154 (24%)	212 (24%)	53 (58%)
	Follow up time median (IQR)	4.2 years (2.82, 4.81)	2.6 years (1.62, 3.68)	3.8 years (2.53, 4.49)	3.7 years (2.33, 4.46)	1.3 years (0.5, 1.5)

*Note: 3 people do not contribute any survival time data.

The majority of patients from the 2002-3 TB Cohort came from the civilian sector, and 19% were prisoners at the time of the study. All patients from this Cohort and the majority of the 2008 XDRTB Cohort patients (96%) were tested for HIV at the point of TB diagnosis; the HIV-positivity rate was higher in the 2008 XDRTB Cohort compared to the 2002-3 TB Cohort patients (9% vs. 4%). None of the HIV-positive patients was receiving highly active antiretroviral treatment (HAART) at the time of the study.

More detailed clinical information was available for the 2008 XDRTB Cohort patients. Approximately half of the XDRTB patients were symptomatic with a productive cough being the most common complaint. SOB (39%), fever (24%) and weight loss (13%) were relatively common but chest pain (13%) and haemoptysis (7%) were rare.

The majority of the 2008 XDRTB Cohort patients had extensive lung damage with more than one segment affected (in 70% of patients) and the presence of cavities in 93% of patients. Almost all patients from this Cohort were smokers (81%); 25% and 46% of them abused alcohol and recreational drugs. Almost all 2008 XDRTB Cohort patients (98%) were hospitalised for treatment. Many patients (44%) were not adherent to anti-tuberculosis therapy and frequent interruptions (for ≤ and ≥1 months (but less than 2 months for qualify for “default”) as well as general poor compliance were reported by attending physicians.

By the end of the follow-up period, 24% of patients in the 2002-3 TB Cohort and 58% in the 2008 XDRTB Cohort had died (Table 1).

### Molecular drug resistance patterns and epidemiology of MDR and XDRTB strains

2002-3 TB Cohort: all available isolates (536/783; 68%) were genotyped; a smaller proportion of strains belonged to the Beijing family among non-MDRTB isolates than for MDRTB isolates where the Beijing family dominated (84% versus 16%; RR 5.1; 95%CI 3.2, 8.2).

2008 XDRTB Cohort: mutations in *katG and inhA* genes associated with isoniazid resistance were detected in 91/92 (98.9%) strains and ninety strains (97.8%) had *rpoB* gene mutations with excellent agreement between molecular and phenotypical DST results (98.9%). Most rifampin-resistant strains had polymorphisms in codon 531 (81 strains, 88.0%). In isoniazid-resistant isolates, 91 harboured the S315T mutation in the *katG* gene which was combined with the mutation C(−15)T in the *inhA* gene in 11 isolates (11.9%).

Sequencing analysis to detect key mutations associated with fluroquinolone resistance was performed on all 92 isolates from the 2008 XDRTB Cohort. Mutations in the QRDR region of the *gyrA* gene were found in 68 (73.9%) isolates with mutations at codon 94 GAC→GGC (D94G) being the most prevalent (57.4%). Other mutations included A90V (16.2%), D94A (16.2%), D94N (4.4%), D94Y (3.0%), and G88A (1.5%). Naturally occurring polymorphisms in codon 95 (AGC-ACC) not associated with resistance to FQ was seen in all but two strains (both belonging to the T1 family) (Table 2).

**Table 2:** XDR strains molecular genotypes and proportions of common mutations associated with fluroquinolone resistance.

Cluster No.*	VNTR profile** (MIRU2,4,10,16,20,23,24,26,27,31,39,40, ETR-A, -B, -C, VNTR424, 1955, 1982, 2347, 2401, 3171, 3232, 3336, 3690, 4052, 4156, 2163A, 2163B)	Mutations in the gyrA gene, no.(%)						
		90 GTG	94 AAC	94 GGC	94 GCC	94 TAC	88 GCC	Wild type
**1 (N = 36)**	223325153533424448443(12)726296	4 (11.1%)	3 (8.3%)	15 (41.7%)	4 (11.1%)	1 (2.8%)	0	9 (25.0%)
**2 (N = 15)**	223325173533424446443(14)727296	3 (20.0%)	0	6 (40.0%)	1 (6.7%)	1 (6.7%)	0	4 (26.7%)
**3 (N = 3)**	223325153533424448443(14)728296	1 (33.3%)	0	2 (66.6%)	0	0	0	0
**4 (N = 3)**	223325173533424446443(14)722296	0	0	1 (33.3%)	2 (66.6%)	0	0	0
**5 (N = 3)**	223325153533424448443(15)728296	0	0	0	1 (33.3%)	0	0	2 (66.6%)
**6 (N = 3)**	223325173533424446443(12)722296	1 (33.3%)	0	1 (33.3%)	1 (33.3%)	0	0	0

*Clusters were identified using VNTR and spoligotyping.

**The VNTR types are given as 28-digit codes corresponding to the numbers of copies of minisatellites in respective loci (please see column heading).

VNTR typing demonstrated that the majority of XDRTB strains (85/92, i.e. 92%) belonged to the Beijing family; other identified families included Haarlem 3 (3 isolates), Family 33 (1), T1 (2), and T4 (1). We showed high clonality of XDRTB strains in Samara with a small number of dominant strains (Table 2).

### Long-term survival and factors associated with survival

#### 2002-3 TB Cohort

**Figure 1:**
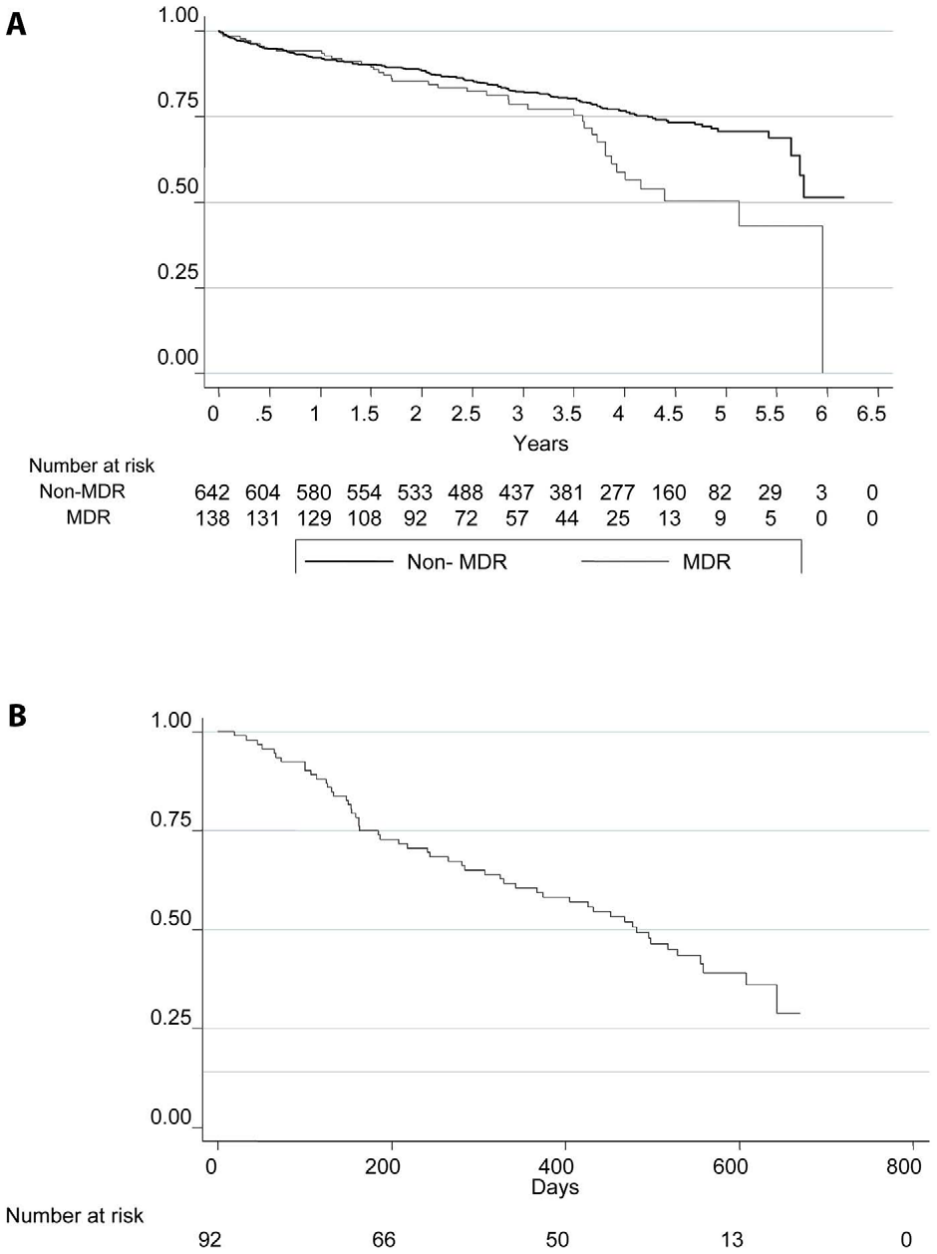
Kaplan-Meier survival curves for patients from (A) 2002-3 TB Cohort defined by MDR- vs non-MDR status and (B) 2008 XDRTB Cohort.

Half (50%) of MDRTB patients and the majority of non-MDR patients (71%) were still alive at 5 years (Figure 1A).

In a univariate Cox regression model for patients from this Cohort (Table 3), survival was significantly associated with being male, older age, MDRTB status, HIV co-infection and being a relapse case. Prisoners were no more likely to die than civilians (HR 1.04; 95% CI 0.75, 1.45). Infection with Beijing family strains had no effect on survival (HR 0.78; 95%CI 0.54, 1.11).

**Table 3:** Uni- and multivariate Cox regression models of predictors of death.

Possible predictor, no.(%)		Univariate model				Multivariate model#	
		2002-3 TB Cohort*		2008 XDRTB Cohort		2002-3 TB Cohort	
		HR (95%CI)	p-value	HR (95%CI)	p-value	HR (95%CI)	p-value
		N = 877		N = 92		N = 778	
**Gender**	Male	1.0		1		1.0	
	Female	0.71 (0.48, 1.05)	0.083	0.67 (0.37, 1.22)	0.191	0.69 (0.47, 1.03)	0.068
		N = 877		N = 92		N = 778	
**Age**	≤40 y.o.	1.0	0.000	1.0	0.56	1.0	0.005
	>40 y.o	1.02 (1.01, 1.03)		1.01 (0.98, 1.03)		1.01 (1.00, 1.02)**	
		N = 877					
**Residence**	Civilian	1.0		N/A		Not included	
	Prison	1.04 (0.75, 1.45)	0.814				
		N = 875		N = 90			
**Treatment history**	New	1.0		1		1.0	
	Re-treatment$	1.94 (1.29, 2.93)	0.002	1.54 (0.37, 6.34)	0.549	1.61 (1.04, 2.49)	0.032
		N = 877		N = 88		N = 778	
**HIV Status**	Negative	1.0		1		1.0	
	Positive	0.46 (0.21, 1.05)	0.065	1.23 (0.49, 3.11)	0.655	0.54 (0.20, 1.48)	0.235
		N = 535		N = 92			
**Strain family** ∧	Beijing	1.0		1			
	Other	0.78 (0.54, 1.11)	0.167	0.99 (0.39, 2.48)	0.976		
		N = 780				N = 778	
**Resistance status** ∧	MDR	1.0		N/A		1.00	
	Non-MDR	1.60 (1.12, 2.27)	0.009			1.67 (1.17, 2.39)	0.005

*Three people died on the same date as diagnosis and hence do not contribute any time to the analysis.

**In the multivariate Cox model age was taken as a continuous variable.

∧ Patients without available DST results from 2002-3 TB Cohort are excluded from the analysis.

# All variables from univariate analysis significant at 10% significance level are included into multivariate analysis (2002-3 TB Cohort); multivariate analysis was not performed for 2008 XDRTB Cohort as no significant variable were identified at univariate analysis in this group.

$ In 2002-3 Cohort re-treatment cases included relapses only; in 2008 XDRTB Cohort re-treatment cases include relapses, return after default, treatment after failure, chronic patients.

In the multivariate model containing all factors of significance only relapse/past treatment and multi-drug resistance remained associated with lower survival (HR 1.61; 95%CI 1.04, 2.49 and HR 1.67, 95%CI 1.17, 2.39 respectively).

Although older age was significantly associated with increased mortality, the effect was relatively small, i.e. per each year increase in age (HR 1.01; 95%CI 1.00, 1.02) (Table 3).

HIV status did not affect survival of patients from the 2002-3 TB Cohort (as overall this Cohort contains predominantly new drug-sensitive TB cases) with comparable survival for patients who were HIV-infected (79% were alive) versus HIV-negative (69% were alive) at 5 years after diagnosis supporting data from other countries that treatment for drug sensitive TB was as effective for HIV positive patients as HIV negative at the early stages of immunodeficiency [30].

#### 2008 XDRTB Cohort

Over half (58%) of the patients died within two years of establishing a diagnosis of XDRTB (Figure 1B).

For XDRTB patients none of the factors reached statistical significance for survival in a univariate Cox regression model including HIV co-infection (HR 1.23; 95%CI 0.49, 3.11): age, gender, treatment history, or strain family (Table 3). However, median survival time for HIV-infected patients with XDRTB was only 185 days (0.5 years) compared to 496 days (1.4 years) for HIV negative participants.

Additionally analysed clinical factors (presence of cough, haemoptysis, SOB, chest pain, weight loss, fever, cavitations, involvement of multiple lung zones, smoking, alcohol and drug abuse, hospitalisation and adherence to therapy) did not appear to affect survival.

## Discussion

Our drug resistance survey conducted earlier in Samara Oblast provided hope for development of a standardized MDRTB treatment regimen with a successful treatment outcome. This follow-up analysis conducted six years later in the same geographical settings showed alarmingly high rates of XDRTB (11.1%) against the background of a rapidly evolving epidemic of HIV.

Molecular genotyping analysis demonstrated that the overwhelming majority of MDRTB/XDRTB (84% and 92% respectively) isolates belonged to the Beijing TB strain family that is substantially higher than was detected through the earlier study (2001–2002) in the same region (62% of those MDRTB strains belonged to the Beijing strain) [24]. In non-MDRTB strains the proportion of isolates from the Beijing strain family was significantly lower. Circulating strains were extremely homogeneous with the majority of XDRTB cases associated with two main clones in Samara. Detailed molecular epidemiological mapping to detect factors facilitating such successful spread of this family strain across Eastern Europe is currently under way. Factors contributing to drug-resistance development, such as poor adherence to therapy and absence of social support as well as of treatment incentives/motivation, frequent treatment interruption and inadequate therapeutic regimens without reliable SLD DST results, have been described previously for Russian settings [6], [24], [31], [32], [33]. Now despite the initiation of international TB control projects the consequences of relatively low cure rates, limited infection control, increasing availability and use of the second-line drugs for both TB and no-TB infections, and their availability without prescription [34], [35] can be seen.

Previous TB treatment cycles and MDRTB and XDRTB were significant risk factors for mortality. Although not reaching statistical significance overall (possibly due to a relatively small number of HIV-positive cases among the overall group) HIV co-infection did result in higher mortality rates confirming data from other studies [36], [37], [38]. The rate of HIV co-infection which was 4% in 2003 almost doubled, reaching 9% in 2008 showing an alarming trend in the convergence of the two epidemics in the region. Recent preliminary analysis for 2010 has suggested that the rate of TB-HIV co-infection has increased further reaching 16% (data not shown).

Nevertheless long-term survival of MDRTB patients was better than reported previously with half of MDRTB patients alive 5 years after diagnosis. However, XDRTB killed more than half of patients in less than 18 months. Prisoners and civilians with MDRTB had a similar mortality. Although providing hope, this also means that many chronically ill patients survive relatively long periods of time and undergo numerous repetitive cycles of empirical treatment including prolonged hospitalizations and subsequent contact with HIV-infected individuals. In these circumstances, introduction of rapid and reliable molecular screening methods for MDRTB and XDRTB would be of value so that MDRTB/XDRTB patients can be segregated early to interrupt transmission and to initiate treatment promptly. The combined use of traditional phenotypical and molecular approaches allows both accurate and fast laboratory screening and testing of sensitivities to the second-line drugs [7], and appropriate initiation of infection control measures and therapy. Where this has occurred successfully, e.g., Peru, survival has improved [39].

The study had some limitations. We were not able to obtain full clinical and epidemiological information for all patients, e.g., information about treatment regimens was not included into analysis of survival but treatment for patients follows Russian Federal regulations which for MDRTB and XDRTB patients involve therapy tailored to individual drug susceptibility results [18]. Nevertheless delays in establishing effective treatment were and remain common. Due to differences between patients' groups, a direct comparison of survival of MDRTB and XDRTB patients was not conducted. Although the current study represents a heterogeneous population, it provides the first evidence of the extent and the pattern of XDRTB and the evolution of significant rates of HIV co-infection in Samara Oblast as well as describes the chances of long-term survival of civilian and prison patients infected with MDRTB /XDRTB strains with and without HIV co-infection. This study is one of the largest in terms of the cohorts' size and length of follow-up.

As Samara Oblast represents a typical setting in the Russian Federation and in many other FSU countries, the data can be generalized country- and region-wide.

## References

[1] Kim DH, Kim HJ, Park SK, Kong SJ, Kim YS (2008). Treatment outcomes and long-term survival in patients with extensively drug-resistant tuberculosis.. Am J Respir Crit Care Med.

[2] Shah NS, Pratt R, Armstrong L, Robison V, Castro KG (2008). Extensively drug-resistant tuberculosis in the United States, 1993-2007.. JAMA.

[3] Jeon DS, Kim DH, Kang HS, Hwang SH, Min JH (2009). Survival and predictors of outcomes in non-HIV-infected patients with extensively drug-resistant tuberculosis.. Int J Tuberc Lung Dis.

[4] He GX, Xie YG, Wang LX, Borgdorff MW, van der Werf MJ (2010). Follow-up of patients with multidrug resistant tuberculosis four years after standardized first-line drug treatment.. PLoS One.

[5] Migliori GB, Lange C, Girardi E, Centis R, Besozzi G (2008). Extensively drug-resistant tuberculosis is worse than multidrug-resistant tuberculosis: different methodology and settings, same results.. Clin Infect Dis.

[6] Bendayan D, Hendler A, Polansky V, Weinberger M (2010). Outcome of hospitalized MDR-TB patients: Israel 2000–2005..

[7] Lonnroth K, Castro KG, Chakaya JM, Chauhan LS, Floyd K (2010). Tuberculosis control and elimination 2010–50: cure, care, and social development.. Lancet.

[8] Koenig R (2008). Drug-resistant tuberculosis. In South Africa, XDR TB and HIV prove a deadly combination.. Science.

[9] Gandhi NR, Moll A, Sturm AW, Pawinski R, Govender T (2006). Extensively drug-resistant tuberculosis as a cause of death in patients co-infected with tuberculosis and HIV in a rural area of South Africa.. Lancet.

[10] Migliori GB, Sotgiu G, D'Arcy Richardson M, Centis R, Facchini A (2009). MDR-TB and XDR-TB: drug resistance and treatment outcomes.. Eur Respir J.

[11] Wells CD, Cegielski JP, Nelson LJ, Laserson KF, Holtz TH (2007). HIV infection and multidrug-resistant tuberculosis: the perfect storm.. J Infect Dis.

[12] Migliori GB, Centis R, Lange C, Richardson MD, Sotgiu G (2009). Emerging epidemic of drug-resistant tuberculosis in Europe, Russia, China, South America and Asia: current status and global perspectives.. Curr Opin Pulm Med.

[13] Wright A, Zignol M, Van Deun A, Falzon D, Gerdes SR (2009). Epidemiology of antituberculosis drug resistance 2002-07: an updated analysis of the Global Project on Anti-Tuberculosis Drug Resistance Surveillance.. Lancet.

[14] Leimane V, Dravniece G, Riekstina V, Sture I, Kammerer S (2010). Treatment outcome of multidrug/extensively drug-resistant tuberculosis in Latvia, 2000–2004.. Eur Respir J.

[15] (2010). Samara AIDS Center Epidemiological Update.. http://wwwnoaidsru/r/11/.

[16] Samara Oblast Tuberculosis Dispensary (2009). Annual Report on TB Control in Samara Oblast.

[17] Balabanova Y, Ruddy M, Hubb J, Yates M, Malomanova N (2005). Multidrug-resistant tuberculosis in Russia: clinical characteristics, analysis of second-line drug resistance and development of standardized therapy.. Eur J Clin Microbiol Infect Dis.

[18] Ministry of Health of the Russian Federation P Prikaz of the Ministry of Health of the Russian Federation # 109 from 21 March 2003 “On improvement of TB control activities in the Russian Federation”.

[19] Siddiqi S, Rusch-Gerdes S, Alexander H, Drobniewski F, Feldman K (2007). MGIT Procedure Manual..

[20] Kruuner A, Yates MD, Drobniewski FA (2006). Evaluation of MGIT 960-based antimicrobial testing and determination of critical concentrations of first- and second-line antimicrobial drugs with drug-resistant clinical strains of Mycobacterium tuberculosis.. J Clin Microbiol.

[21] World Health Organization (2008). Policy guidance on drug-susceptibility testing (DST) of second-line antituberculosis drugs.. Geneva WHO/HTM/TB/2008392.

[22] Brown TJ, Herrera-Leon L, Anthony RM, Drobniewski FA (2006). The use of macroarrays for the identification of MDR Mycobacterium tuberculosis.. J Microbiol Methods.

[23] Nikolayevskyy VV, Brown TJ, Bazhora YI, Asmolov AA, Balabanova YM (2007). Molecular epidemiology and prevalence of mutations conferring rifampicin and isoniazid resistance in Mycobacterium tuberculosis strains from the southern Ukraine.. Clin Microbiol Infect.

[24] Drobniewski F, Balabanova Y, Nikolayevsky V, Ruddy M, Kuznetzov S (2005). Drug-resistant tuberculosis, clinical virulence, and the dominance of the Beijing strain family in Russia.. JAMA.

[25] Velji P, Nikolayevskyy V, Brown T, Drobniewski F (2009). Discriminatory ability of hypervariable variable number tandem repeat loci in population-based analysis of Mycobacterium tuberculosis strains, London, UK.. Emerg Infect Dis.

[26] Kwara A, Schiro R, Cowan LS, Hyslop NE, Wiser MF (2003). Evaluation of the epidemiologic utility of secondary typing methods for differentiation of Mycobacterium tuberculosis isolates.. J Clin Microbiol.

[27] Nikolayevskyy V, Gopaul K, Balabanova Y, Brown T, Fedorin I (2006). Differentiation of tuberculosis strains in a population with mainly Beijing-family strains.. Emerg Infect Dis.

[28] Gopaul KK, Brown TJ, Gibson AL, Yates MD, Drobniewski FA (2006). Progression toward an improved DNA amplification-based typing technique in the study of Mycobacterium tuberculosis epidemiology.. J Clin Microbiol.

[29] Balabanova Y, Drobniewski F, Fedorin I, Zakharova S, Nikolayevskyy V (2006). The Directly Observed Therapy Short-Course (DOTS) strategy in Samara Oblast, Russian Federation.. Respir Res.

[30] Murray J, Sonnenberg P, Shearer SC, Godfrey-Faussett P (1999). Human immunodeficiency virus and the outcome of treatment for new and recurrent pulmonary tuberculosis in African patients.. Am J Respir Crit Care Med.

[31] Atun RA, Samyshkin YA, Drobniewski F, Kuznetsov SI, Fedorin IM (2005). Seasonal variation and hospital utilization for tuberculosis in Russia: hospitals as social care institutions.. Eur J Public Health.

[32] Drobniewski F (1995). Tuberculosis in prisons--forgotten plague.. Lancet.

[33] Ruddy M, Balabanova Y, Graham C, Fedorin I, Malomanova N (2005). Rates of drug resistance and risk factor analysis in civilian and prison patients with tuberculosis in Samara Region, Russia.. Thorax.

[34] World Health Organization (2008). Global tuberculosis control: surveillance, planning, financing.. Geneva.

[35] Balabanova Y, Fedorin I, Kuznetsov S, Graham C, Ruddy M (2004). Antimicrobial prescribing patterns for respiratory diseases including tuberculosis in Russia: a possible role in drug resistance?. J Antimicrob Chemother.

[36] Gandhi NR, Shah NS, Andrews JR, Vella V, Moll AP (2009). HIV Co-infection in Multidrug- and Extensively Drug-resistant Tuberculosis Results in High Early Mortality.. Am J Respir Crit Care Med.

[37] Podlekareva DN, Mocroft A, Post FA, Riekstina V, Miro JM (2009). Mortality from HIV and TB coinfections is higher in Eastern Europe than in Western Europe and Argentina.. AIDS.

[38] Dheda K, Warren RM, Zumla A, Grobusch MP (2010). Extensively drug-resistant tuberculosis: epidemiology and management challenges.. Infect Dis Clin North Am.

[39] Mitnick CD, Shin SS, Seung KJ, Rich ML, Atwood SS (2008). Comprehensive treatment of extensively drug-resistant tuberculosis.. N Engl J Med.

